# Concurrent Scleredema and Pyoderma Gangrenosum: Case Report and Review of Comorbid Conditions

**DOI:** 10.7759/cureus.12188

**Published:** 2020-12-20

**Authors:** Joanne S Jacob, Philip R Cohen

**Affiliations:** 1 Medicine, Baylor College of Medicine, Houston, USA; 2 Dermatology, San Diego Family Dermatology, National City, USA

**Keywords:** arthritis, bowel, diabetes, disease, gammopathy, gangrenosum, inflammatory, myeloma, pyoderma, scleredema

## Abstract

Scleredema is a connective tissue disorder that presents as diffuse induration of skin, most often involving the upper body. Scleredema can be associated with prior infection, monoclonal gammopathy, and diabetes mellitus. Pyoderma gangrenosum is a neutrophilic dermatosis that presents as an ulcer with violaceous borders. Pyoderma gangrenosum can be idiopathic or associated with various conditions. A 66-year-old man with a 20-year history of scleredema diabeticorum presented with idiopathic pyoderma gangrenosum in the affected area of scleredema on his neck. His pyoderma gangrenosum resolved after treatment with topical and intralesional corticosteroids. Diseases associated with scleredema, pyoderma gangrenosum or both are reviewed.

## Introduction

Scleredema is a connective tissue disease characterized by symmetric and progressive induration classically affecting the posterior neck and upper back. Prior infection, monoclonal gammopathy, and diabetes mellitus have been associated with scleredema. Multiple treatment modalities with variable success have been described [1].

Pyoderma gangrenosum is a neutrophilic dermatosis; it has several clinical variants and the classical presentation of pyoderma gangrenosum is an ulcer with overhanging violaceous borders. Various conditions have been associated with pyoderma gangrenosum; however, the dermatosis can also be idiopathic. Treatment of disease-associated pyoderma gangrenosum is typically directed toward the underlying etiology [2].

A 66-year-old man who had a 20-year history of scleredema diabeticorum, presented with idiopathic pyoderma gangrenosum in the affected areas of scleredema on his posterior neck. The ulcerative pyoderma gangrenosum lesion eventually resolved completely after treatment with topical and intralesional corticosteroids. Diseases associated with scleredema, pyoderma gangrenosum or both are reviewed.

## Case presentation

**Figure 1:**
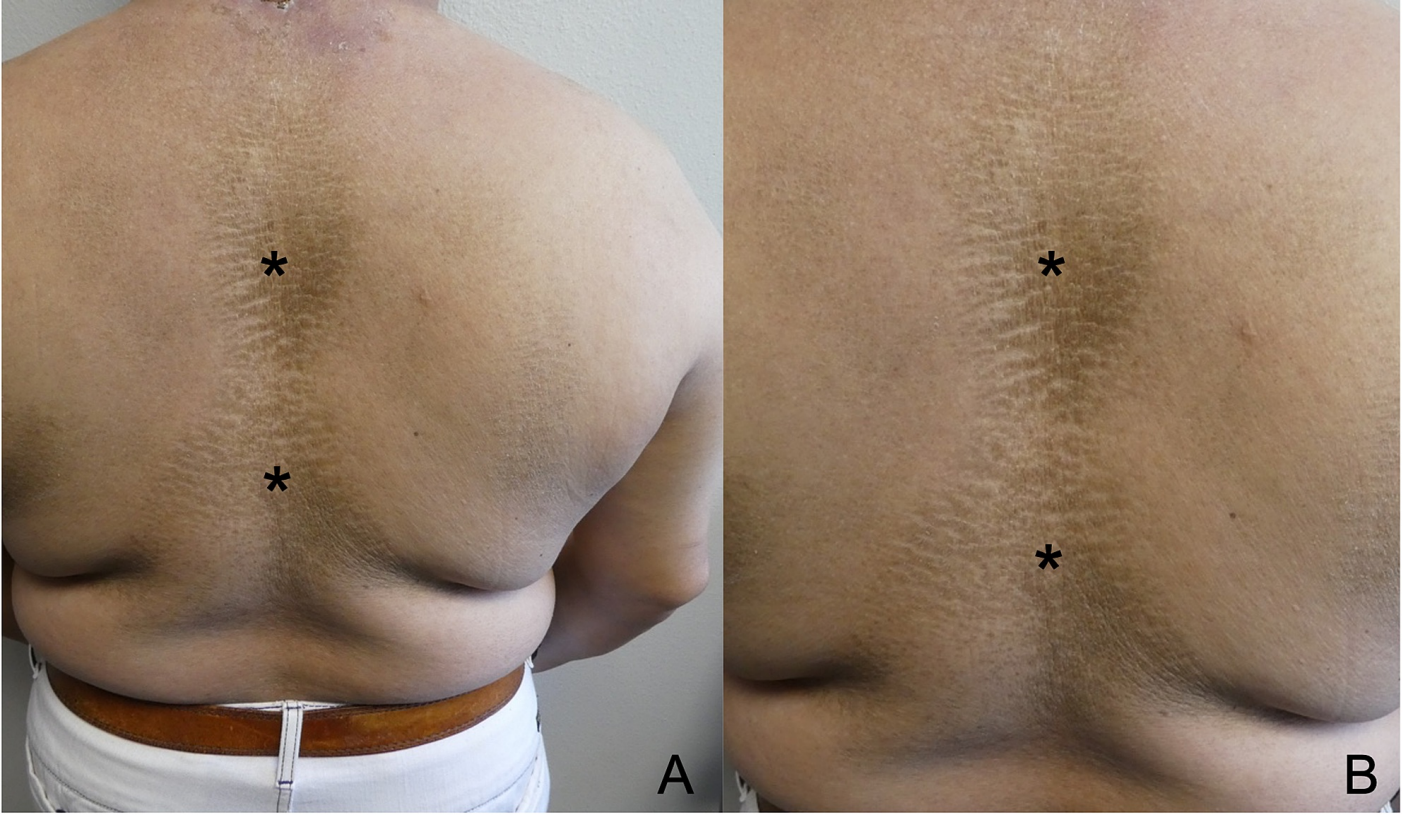
Scleredema of the back Distant (A) and closer (B) views of the scleredema diabeticorum of the upper back of a 66-year-old man with a 20-year history of diabetes. His scleredema appeared as chronic brown hyperkeratosis (black asterix) and induration on his central upper back; similar clinical findings were also present on his posterior neck.

A 66-year-old man with scleredema diabeticorum presented with an ulcer on the posterior neck. He had a 20-year history of diabetes and a chronic brown hyperkeratosis with underlying induration on his central upper back and posterior neck. Correlation of his previous skin biopsies and the history of diabetes established the condition as scleredema diabeticorum (Figure 1).

**Figure 2:**
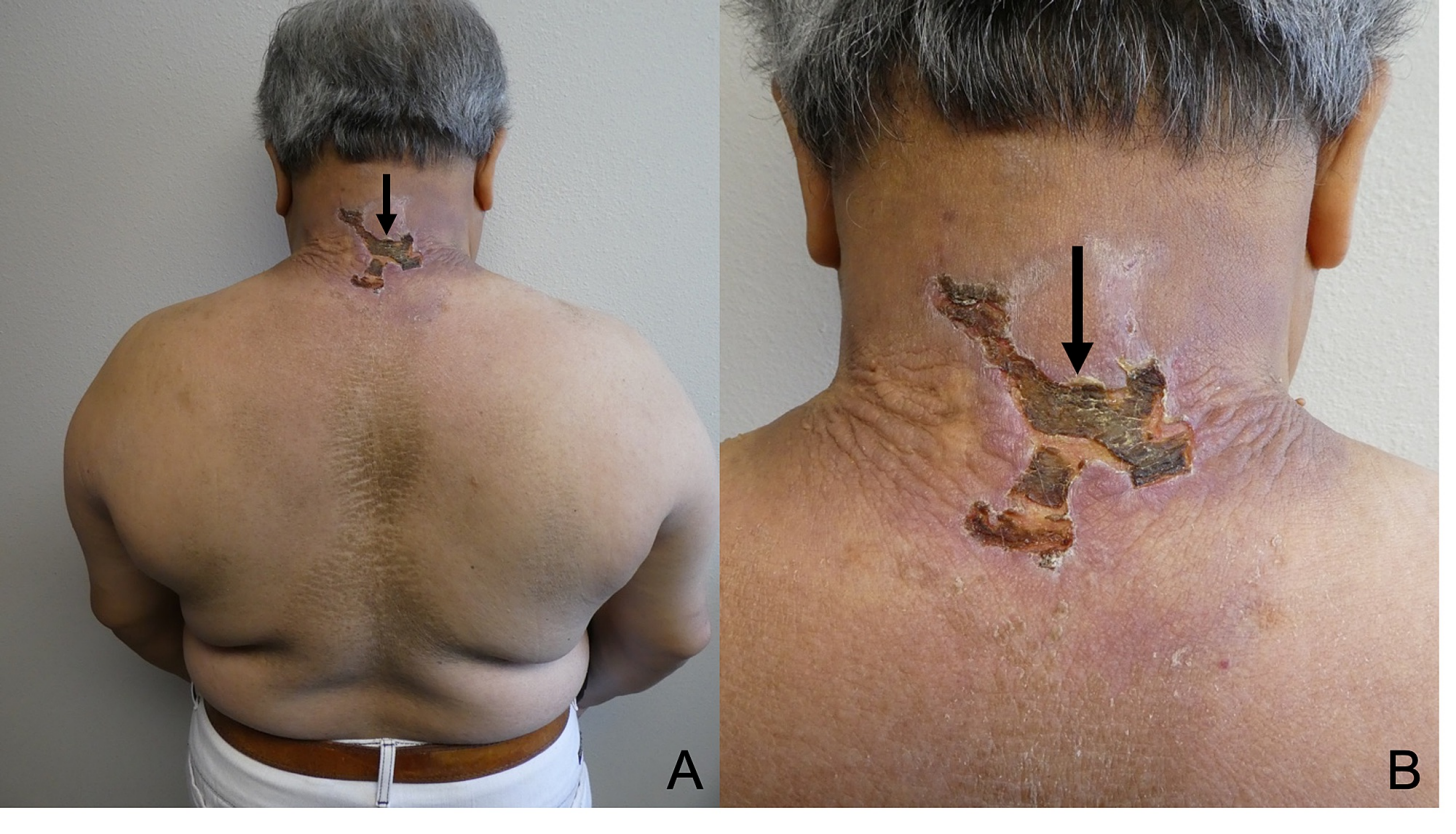
Pyoderma gangrenosum of the posterior neck Distant (A) and closer (B) views of the posterior neck of a man with scleredema diabeticorum that affected his upper back and neck. Pyoderma gangrenosum on his posterior neck presented as an ulcer (black arrow) that measured five by five centimeters in greatest diameter; the ulcer had overhanging edges that extended toward the wound center.

A few weeks before he presented for evaluation, an enlarging, tender ulcer developed. It appeared on an area involved with scleredema -- his posterior neck. The edges of the ulcer overhung toward the ulcer center (Figure 2).

He was started on oral double-strength trimethoprim-sulfamethoxazole and topical mupirocin 2% ointment twice daily for two weeks for the ulcer, and triamcinolone 0.1% cream twice daily for the underlying scleredema. The bacterial culture of the ulcer grew skin flora. The antibiotic was stopped after four weeks of treatment.

After two weeks, there was no improvement in the scleredema. The topical triamcinolone cream was discontinued. The ulcer persisted; therefore, a punch biopsy of the ulcer edge was performed.

Microscopic examination of the posterior neck specimen showed epithelial hyperplasia and deep dermal fibrosis. The dermis contained granulation tissue and telangiectasia with chronic inflammation. Special stains were negative for bacteria, fungi, and mycobacteria. Bacterial, fungal, and mycobacterial skin biopsy cultures were also negative.

Several other lab examinations were conducted. A comprehensive metabolic panel and thyroid-stimulating hormone were normal. Total iron and iron saturation were low with a normal iron-binding capacity. Hemoglobin A1c was elevated to 8.6% (normal, less than 5.7%). A complete blood count showed normocytic anemia with an elevated red blood cell distribution width; prothrombin and partial thromboplastin time were in the normal range. Tests for antineutrophil cytoplasmic antibodies, anti-streptolysin, hepatitis B, hepatitis C, human immunodeficiency virus, glucose-6-phosphate dehydrogenase deficiency, syphilis, and tuberculosis were all negative.

Serum protein electrophoresis showed a slight elevation of alpha 2 globulin, consistent with acute inflammation. Urine protein electrophoresis showed elevated total protein, but no abnormal protein bands were detected. Antinuclear antibody was mildly elevated at a titer of 1:40 (normal, less than 1:40) with a homogenous pattern; anti-Smith antibody, anti-ribonuclear protein antibody, chromatin antibody, double-stranded DNA antibody, Jo-1 antibodies, SCL-70 antibody, Sjogren syndrome antibody A, and Sjogren syndrome antibody B were negative. A colonoscopy, one year earlier, was also normal.

Correlation of the history, lesion morphology, pathologic changes, negative cultures, and laboratory studies established the diagnosis of idiopathic pyoderma gangrenosum. Intralesional triamcinolone (2 milliliters of 10 milligrams/milliliter) was injected into the ulcer edge. Topical betamethasone 0.05% cream was initiated for use twice daily.

**Figure 3:**
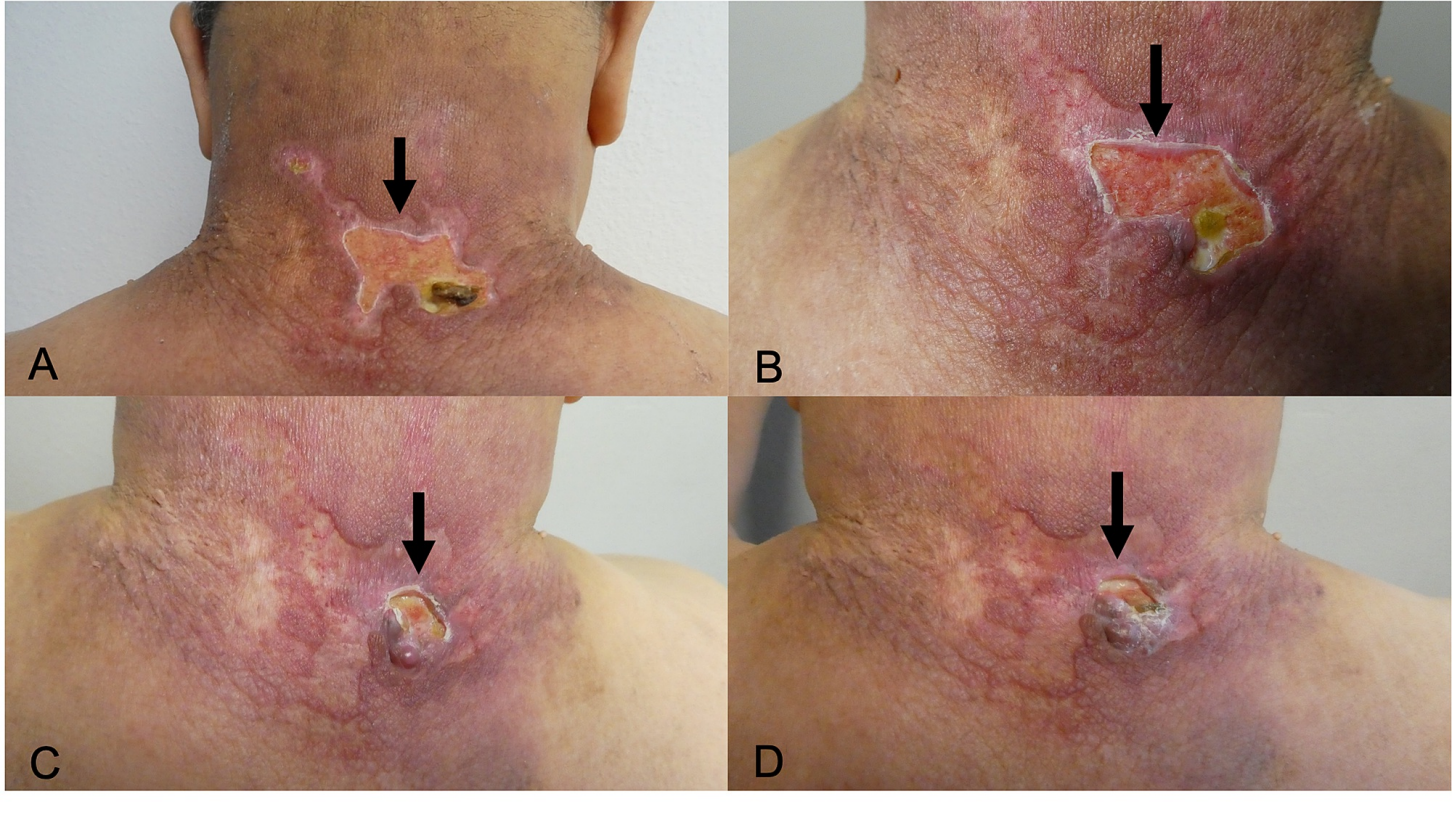
Progressive improvement of pyoderma gangrenosum The sequential images (A, B, C, and D) show improvement of the posterior neck pyoderma gangrenosum. Progressive healing of the ulcer (black arrow) is demonstrated five weeks (A), 10 weeks (B), 24 weeks (C), and 29 weeks (D) after starting intralesional triamcinolone every two weeks and topical betamethasone 0.05% cream twice daily. The initial concentration of triamcinolone was 10 milligrams per milliliter; after 14 weeks of treatment, the dose was increased to 20 milligrams per milliliter in an attempt to expedite the clinical improvement. At each treatment session, he received either 1 milliliter or 2 milliliters of triamcinolone.

Follow-up was continued at two-week intervals for seven months with repeat administration of 1-2 milliliters of intralesional triamcinolone; during treatment, the concentration of intralesional triamcinolone was increased to 20 milligrams/milliliters. A decrease in the size of the ulcer was noticed at each subsequent appointment (Figure 3).

His final intralesional triamcinolone treatment was at 29 weeks. A lapse in intralesional triamcinolone treatment resulted from an office closure and limited on-site patient visits because of the Covid pandemic. He continued the twice daily application of betamethasone 0.05% cream for a total of six months.

**Figure 4:**
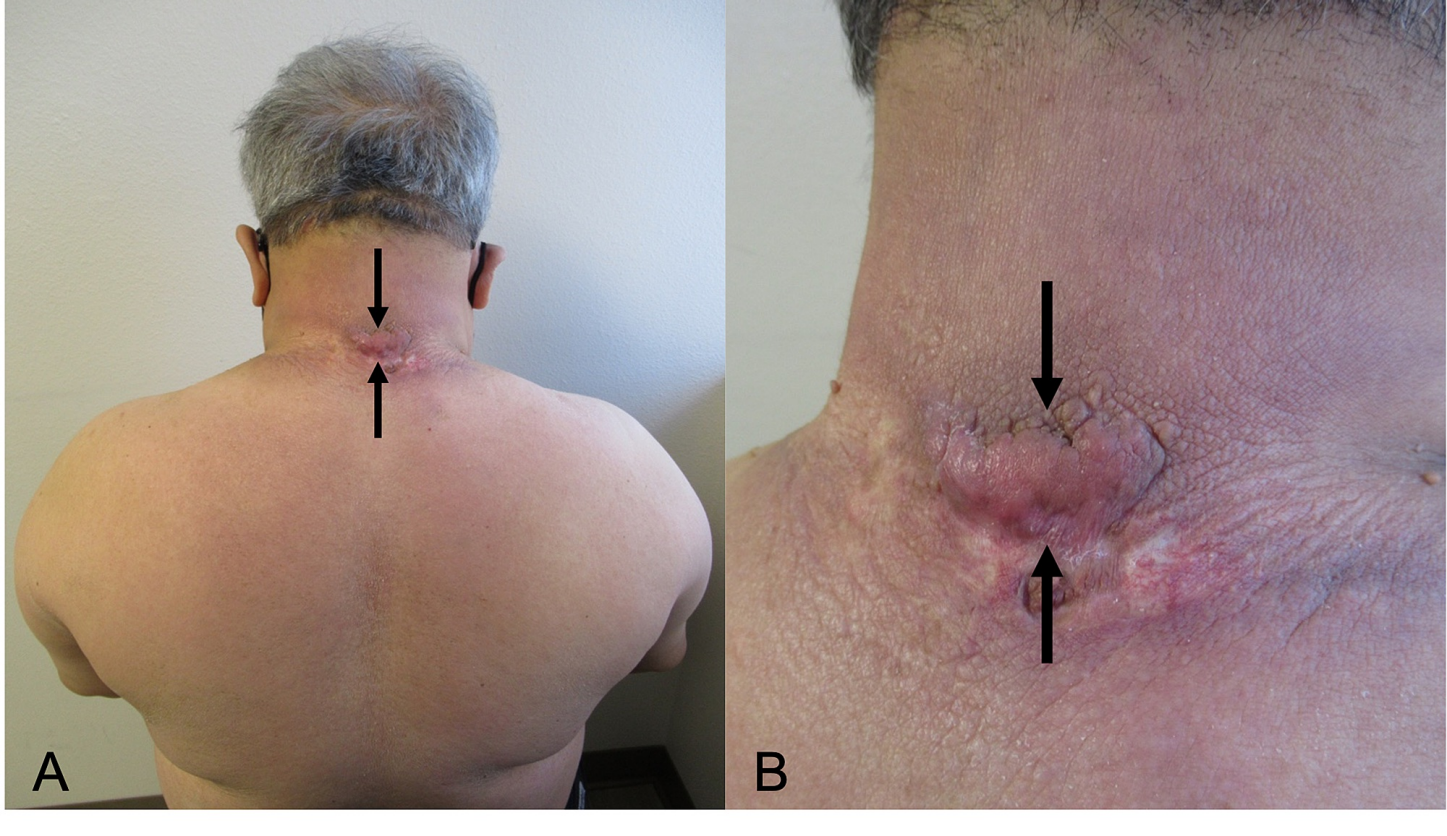
The pyoderma gangrenosum has resolved and the scleredema diabeticorum persists Distant (A) and closer (B) views of the patient’s back and posterior neck at a follow-up examination 14 months after his initial office visit. Eight months following the initial presentation of pyoderma gangrenosum, the ulcer had completely resolved. A hypertrophic scar (between the black arrows) developed at the location of the superior border of the ulcer.

The ulcer subsequently healed. He stopped using the betamethasone 0.05% cream four weeks after the complete resolution of his pyoderma gangrenosum. At subsequent follow-up, the pyoderma gangrenosum ulcer had completely resolved and focally there was a hypertrophic scar (Figure 4).

## Discussion

Scleredema is a skin disease characterized by fibrous, woody induration, occurring most often on the upper body. It most often presents on the neck and extends to the shoulders and back; the face and scalp can also be affected. The clinical differential diagnoses of scleredema include dermatoses characterized by skin thickening, mucin deposition, or both; these conditions include cardiac or renal edema, cutaneous amyloidosis, eosinophilic fasciitis, lymphedema, myxedema, scleromyxedema, and systemic sclerosis [1,3,4].

The histopathology of scleredema typically reveals thickening of the dermis and increased interstitial mucin deposition. The dermis can be up to four times thicker than the dermis found in the normal adjacent tissue. Either an Alcian blue stain or a colloidal iron stain will reveal mucin between collagen fibers of the thickened dermis [1,3]. The diagnosis of scleredema is established by correlating the clinical appearance of the affected skin to these pathologic findings [4].

Scleredema can be idiopathic, or it can be one of three recognized types of scleredema; each is associated with a different comorbid condition. Type one occurs after an infection, typically bacterial or viral, and resolves in a few weeks. Type two is associated with paraproteinemia; it has a slower and more chronic course. Type three, also known as scleredema diabeticorum, is associated with diabetes mellitus and has a progressive, chronic course [1,5,6].

Paraproteinemias associated with scleredema include hypergammaglobulinemia and multiple myeloma. A 34-year-old man with hypergammaglobulinemia and B-cell lymphoma was found to have tightness and thickening of the skin on the chest; the diagnosis of scleredema was established by skin biopsy and he was treated with prednisone [7]. Another example of paraprotein-associated scleredema is a 66-year-old woman with immunoglobulin A-k smoldering myeloma who was found to have scleredema; after a skin biopsy confirmed the diagnosis of scleredema, chemotherapy was initiated for her myeloma and the diffuse thickening of her skin improved [3].

However, comorbidities outside of these three types have also been observed in patients with scleredema; comorbid conditions are summarized in Table 1 [1,5-10]. Some of the conditions include thyroid disease (hyperthyroidism), autoimmune disorders (Sjogren syndrome), and malignancy [8,9]. A 30-year-old man with tear production impairment and salivary gland scintigraphy consistent with Sjogren syndrome was also found to have diffuse induration of the skin of the shoulders and upper arms; the diagnosis of scleredema was made by histopathologic examination [8].

**Table 1:** Comorbid conditions of scleredema ^a^Frequencies for the occurrence of comorbid conditions are cited from the case series from Rongiolietti et al. [9]. Many of the conditions in the miscellaneous category are reported as single case reports.

Comorbid condition	Reported frequency^a^	References
Diabetes mellitus	55%	[1]
Infection	4%	[1,5]
Idiopathic	11%	[6]
Paraproteinemia	11%	[3,6,7]
Miscellaneous		
Autoimmune disorder	Single case	[8]
Malignancy	2%	[9]
Pregnancy	Single case	[10]
Pyoderma gangrenosum	Single case	[Current report]
Thyroid disease	16%	[9]

Individual reports of scleredema patients with hematologic malignancy (chronic myeloid leukemia) have been described. A patient with chronic myeloid leukemia had skin lesions that resolved after treatment with systemic corticosteroids and imatinib [9]. Other patients with scleredema had solid tumors of the thyroid and breast [9].

In addition, scleredema has been observed in a pregnant 24-year-old pregnant woman who was found to have diffuse thickening of the skin of the abdomen and thighs. One week prior, she had an upper respiratory infection. A biopsy of the affected skin on her thigh showed pathologic features of scleredema and she was treated with amoxicillin-clavulanic acid for two weeks. Her scleredema resolved three months after delivery [10].

Successful treatment of scleredema has been demonstrated with various agents. Topical and intralesional triamcinolone has been shown to improve skin lesions. Systemic methotrexate and cyclosporine have resulted in complete resolution of scleredema. Frequency-modulated electromagnetic neural stimulation, which has been used for diabetic neuropathy, has also demonstrated efficacy in scleredema diabeticorum. In some patients, scleredema has resolved spontaneously [1,11].

Pyoderma gangrenosum is a neutrophilic dermatosis that presents with painful, erythematous ulcers. The classic presentation is a papule or nodule that breaks down into an ulcer with a violaceous border. The clinical differential diagnosis for pyoderma gangrenosum includes not only cutaneous bacterial or fungal infections, but also vasculitis [2].

Histologic features of pyoderma gangrenosum include suppurative inflammation of the dermis. Biopsies performed in the early stages of the disease may reveal neutrophilic infiltration of the dermis. Staining and cultures may be helpful to exclude similar-appearing infectious processes; however, a secondary bacterial infection of impetiginization may be present [2,12]. The diagnosis of pyoderma gangrenosum is established using clinical appearance, clinical history, histopathologic findings, and exclusion of infection [13].

Many diseases can be associated with pyoderma gangrenosum; these are summarized in Table 2 [12,14-19]. The most common is inflammatory bowel disease, which has been found in two-thirds of patients with pyoderma gangrenosum. In these individuals, pyoderma gangrenosum is often diagnosed first, leading to earlier detection of inflammatory bowel disease [14,15].

**Table 2:** Comorbid conditions of pyoderma gangrenosum ^a^Frequencies for the comorbid conditions are cited from the case series by Ashchyan et al. [14]. Many of the conditions in the miscellaneous category are single case reports. ^b^Syndromes include pyoderma gangrenosum, acne, and pyogenic sterile arthritis (PAPA syndrome); pyoderma gangrenosum, acne, and hidradenitis suppurativa (PASH syndrome); pyogenic arthritis, pyoderma gangrenosum, acne, and suppurative hidradenitis (PAPASH syndrome); psoriatic arthritis, pyoderma gangrenosum, acne, and suppurative hidradenitis (PsAPASH syndrome); and pyoderma gangrenosum, acne, suppurative hidradenitis, and ankylosing spondylitis (PASS syndrome).

Comorbid condition	Reported frequency^a^	References
Arthritis	21%	[14,15]
Hematologic disorders	11%	[14,16]
Idiopathic	33%	[14]
Inflammatory bowel disease	41%	[14,15]
Miscellaneous		
Diabetes mellitus	Single case	[17]
Pregnancy	Single case	[18]
Scleredema	Single case	[Current report]
Syndromes^b^	Not reported	[12,19]

Arthritis is the second most common disease associated with pyoderma gangrenosum. It can occur in the setting of pyoderma gangrenosum-associated inflammatory bowel disease. In addition, arthritis can present as a seronegative idiopathic condition in patients with pyoderma gangrenosum [14,15].

Retrospective studies have also demonstrated an association between pyoderma gangrenosum and hematologic malignancies. These include leukemia (such as acute myelogenous leukemia and chronic myelogenous leukemia) and non-Hodgkin lymphoma. In addition, monoclonal gammopathy and myelodysplastic syndrome have also been associated with pyoderma gangrenosum [14,16]. 

Pyoderma gangrenosum has also been observed in a patient with diabetes. A 55-year-old man with type 2 diabetes was found to have a necrotic, purulent ulcer on the lower extremity. Though initially thought to be a diabetic ulcer, clinical features and histopathology pointed towards a final diagnosis of pyoderma gangrenosum [17].

Pyoderma gangrenosum has also been reported in association with pregnancy. A 28-year-old woman developed an ulcer in the perineal region. Biopsy displayed histologic features consistent with pyoderma gangrenosum. Oral prednisolone was started, but the ulcer was still present at the end of her pregnancy. A cesarean section was performed, and the perineal ulcer resolved one month later [18].

There are also several syndromes in which pyoderma gangrenosum is an associated component. One is an autosomal dominant condition that presents with pyoderma gangrenosum, acne, and pyogenic sterile arthritis (PAPA syndrome). Another syndrome presents with pyoderma gangrenosum, acne, and hidradenitis suppurativa (PASH syndrome). A third syndrome is characterized by pyogenic arthritis, pyoderma gangrenosum, acne, and suppurative hidradenitis (PAPASH syndrome). A fourth syndrome presents with psoriatic arthritis, pyoderma gangrenosum, acne, and suppurative hidradenitis (PsAPASH syndrome). Finally, a fifth syndrome presents with pyoderma gangrenosum, acne, suppurative hidradenitis, and ankylosing spondylitis (PASS syndrome) [12,19].

There are several options for the management of pyoderma gangrenosum. Immunosuppressants such as corticosteroids or cyclosporine are often the initial agent, used either alone for limited disease or in a combination with other agents for individuals with severe or refractory disease. Slower-acting biologics such as adalimumab, etanercept, and infliximab have been used as supplemental agents [12,20].

Pyoderma gangrenosum occurring in the same location as scleredema, to the best of our knowledge, has not been described. Both diseases can be associated with monoclonal gammopathy [3,14]. Single patients with other conditions in common include pregnancy and chronic myeloid leukemia [9,10,16,18]. Diabetes has also been described in pyoderma gangrenosum patients; however, this may be a coincidental finding and not related to neutrophilic dermatosis [17].

## Conclusions

Scleredema and pyoderma gangrenosum are distinct conditions with various comorbid diseases. Scleredema has been reported in association with chronic myeloid leukemia, diabetes mellitus, hyperthyroidism, infection, paraproteinemia, pregnancy, Sjogren syndrome, and solid tumors. Pyoderma gangrenosum has been associated with arthritis, diabetes mellitus, inflammatory bowel disease, genetic syndromes, hematologic malignancies, monoclonal gammopathy, myelodysplastic syndrome, and pregnancy. Of these, chronic myeloid leukemia, monoclonal gammopathy, and pregnancy have been found in both conditions. Our patient had a 20-year history of scleredema diabeticorum and presented with a new ulcer. An extensive evaluation including not only his clinical history, the lesion appearance, and the biopsy pathology findings, but also the negative tissue cultures and laboratory studies established the diagnosis of pyoderma gangrenosum. The ulcer eventually resolved after treatment with intralesional triamcinolone and topical betamethasone. Albeit rare, concurrent scleredema and pyoderma gangrenosum -- as seen in our patient -- may occur.
